# Unique cessation tools in the box: Quitline utilization and effectiveness trends among a large sample of tobacco users reporting mental health disorders

**DOI:** 10.3389/fpsyt.2022.869802

**Published:** 2022-07-19

**Authors:** Jonathan T. Hart, Lindsay M. Boeckman, Laura A. Beebe

**Affiliations:** ^1^Stephenson Cancer Center and Department of Pediatrics, University of Oklahoma Health Sciences Center, Oklahoma City, OK, United States; ^2^Hudson College of Public Health, University of Oklahoma Health Sciences Center, Oklahoma City, OK, United States

**Keywords:** quitline, tobacco cessation, mental health, smoking, stress

## Abstract

It is estimated that the prevalence of smoking among adults with MHDs ranges between 40-60%, as compared to about 17% among those without an MHD. In addition, smokers with MHDs smoke more cigarettes, are more nicotine dependent, and experience more difficulty quitting, compared to other smokers. The uniquely high smoking prevalence among the MHD population is a serious public health concern; unfortunately, a majority of individuals experiencing difficulty receive no treatment. The US Public Health Service guidelines, as well as the National Cancer Institute, strongly recommend quitlines as an evidence-based treatment strategy to reduce barriers to cessation treatment, especially among smokers with MHDs; however, the literature is sparse on quitline engagement trends and associated outcomes for quitline participants with MHDs. This study sought to contribute to this gap with the largest sample to-date of MHD-endorsing tobacco quitline (Oklahoma Tobacco Helpline, OTH) participants. From 2015 to 2020, ~65,000 registrants (45-50% of total registered participants) with the OTH identified as having one or more MHDs in addition to their tobacco use. This study tested for the presence of significant differences between groups with and without MHDs (as well as within the MHD-identified group) on program enrollment selections, the intensity of engagement with chosen services, NRT utilization, and quit rates. It also tested for the existence of differences and moderating effects of demographic variables associated with the comparison groups. Statistically significant differences were found between these two groups with regard to: sex, age, racial identity, education level, annual income and insurance status. Significant differences were also found with tobacco use patterns reported by individuals (e.g., timing and daily use amounts). Differences in quitline program selection were demonstrated, such that the MHD-endorsing sample were more likely to participate and agree to the most robust service available. Significantly higher rates of service intensity (number of services engaged) were demonstrated, and MHD individuals were also significantly more likely to receive NRT as a part of their treatment. This study suggests a simplistic “more is better” quitline services approach may suffer in effectiveness because it neglects barriers common to this population. Important information is provided on these unique variables associated with MHD-endorsing individuals trying to quit their tobacco use. These results can help tobacco quitlines conceptualize the unique difficulties experienced by individuals with MHDs and then tailor their approach to respond supportively and constructively to this high need group.

## Introduction

There are an estimated 52.9 million adults (21.0% of the adult population) suffering from mental health disorders (MHDs) in the United States (1). Among those with mental health disorders, ~17 million are also diagnosed with a co-occurring substance use disorder (SUD) and frequently also present with co-morbid physical health conditions (1–3). A recent review revealed that only 7.4% of these individuals receive treatment for both disorders, while 55% receive no treatment at all (4). Of the substances typically abused by Individuals with mental health disorders, tobacco is one of the most common (5). It is estimated that the prevalence of smoking among adults with MHDs ranges between 40-60%, as compared to about 17% among those without any mental health conditions (6). Furthermore, smokers with MHDs tend to smoke more cigarettes, be more nicotine dependent, and experience more difficulty quitting as compared to smokers without co-occurring MHDs (7, 8). While overall smoking rates have declined in recent years, rates among those with MHDs have remained almost the same, with about 45% of annual tobacco-related deaths estimated to be among smokers with MHDs (9, 10). The uniquely high smoking prevalence among this population should be cause for serious concern representing a significant public health disparity.

As noted above, an estimated 55% of individuals with a co-occurring mental health and substance abuse disorders (to include nicotine dependence) receive no treatment. For those who do receive treatment, it is often not for both disorders present (i.e., treatment targets mental health-related symptoms but doesn't address nicotine dependence). Barriers to treatment for this group in particular have been well-documented and include: physical access to treatment sites, healthcare, time and financial burden, etc. (e.g., lack of paid time off for medical appointments) (11). One unique treatment that overcomes several of these noted barriers and has been demonstrated to be effective with smokers (including smokers with MHDs) are state tobacco quitline services (12, 13).

The US Public Health Service guidelines, as well as the National Cancer Institute, strongly recommend quitlines as a treatment strategy to reduce barriers to cessation treatment, especially among smokers with mental health disorders. Specifically, quitlines represent an endorsed best practices approach with their provision of both cessation coaching and supporting nicotine replacement therapy typically represented in their multiple call program protocols (14, 15). Quitlines are available in all 50 states and two US territories and provide confidential, free cessation counseling by trained staff in multiple languages to ~400,000 smokers each year. Most quitlines also provide free nicotine replacement therapy (NRT) in the forms of nicotine patches, gum, and lozenges in durations from 2 weeks of mono-NRT up to 12 weeks of combination NRT (16). It has been found that about half of the callers to state quitlines report having at least one MHD; however, this group of quitline callers tend to have lower reported rates of quitting as compared to callers without any MHDs (17, 18).

Efforts to understand and address this disparity have included examination of unique variables and quitline trends associated with quitline callers identifying with MHDs, as well as development of tailored quitline protocols (11–13, 19). Implemented enhancements included unique cessation counseling strategies and coach training, access to a greater number of counseling calls, and access to more weeks of nicotine replacement therapy (NRT) to support a quit attempt (12, 20). The literature is limited on quitline engagement trends and associated outcomes of quitline participants with MHDs. One study examining a group of three states' participants in 2012–13 found that individuals with reported mental health conditions enrolled in a multiple call program tended to complete more calls than individuals without mental health conditions; however, they were less likely to receive NRT from the quitline (21). Another study found that participants with MHDs were more likely to choose a combination of coaching calls and NRT compared to a sample of participants without MHDs (22).

More research is needed to establish a sufficient evidence base to determine which adaptions of current quitline services actually improve quitline effectiveness with this unique population. Undoubtedly, this quest is made more difficult in part due to the fact that this population is not a homogenous group outside of their identification with experiencing an MHD (a diverse category within itself as well). Individuals within the MHD population differ on demographic variables also known to have unique correlations to smoking status such as age, race, sex, SES, geographic location, and co-morbid physical health conditions (23, 24). Also, significant differences between disorders exist, such as Schizophrenia and Attention-Deficit/Hyperactivity Disorder or Bipolar Disorder and Adjustment Disorder. For example, one study demonstrated anxiety disorders in particular can be uniquely problematic with regards to tobacco cessation success, and that individuals struggling with anxiety could benefit from an approach unique to their specific difficulty (not unlike the myriad adaptations of cognitive-behavioral therapy) (25, 26).

Although the heterogeneity within this group is significant, good argument can still be made for the importance of identifying cross-cutting common variables to help inform tailored quitline adaptations. As state quitlines seek to tailor their services to magnify their impact with priority groups, more service options are being made available and are demonstrating effectiveness (e.g., Behavioral health quitline programs, text- and web-based live interactions, as well as automated options) (12, 27, 28). Quitline use trends and associated outcomes are needed data to help state quitlines prioritize limited service delivery resources and guide targeted marketing dedicated to promotion of cessation support and programming.

Oklahoma has one of the highest prevalence rates of smoking in the country, with the most recent estimates suggesting a smoking prevalence rate among adults in the state to be 19.1%, compared to a national rate of 17.1% (14). The Oklahoma Tobacco Helpline (OTH) has been in operation since 2003 and serves ~25,000 individuals each year; it has consistently been ranked in the top five quitlines for reach across North America (per the North American Quitline Consortium). Based on annual internal survey data, between 2015 and 2020, ~65,000 registrants (45–50% of total registered participants) with the OTH identified as having one or more MHDs in addition to their tobacco use.

The purpose of this analysis is to examine quitline service use and engagement trends in a large, recent sample of participants with and without reported MHDs. Differences between groups (with and without MHDs) as well as within the MHD-identified group are explored to identify significant differences in program enrollment selections, the intensity of engagement with chosen services, NRT utilization, and quit rates.

Specifically, the following research questions were examined for a sample of OTH participants eligible for the multiple call program (5 calls and at least 2 weeks of combination NRT):

- Among those eligible for the multiple call program, do significant demographic differences exist between groups with and without MHD endorsement?
- Do individuals endorsing MHDs demonstrate a pattern of Helpline service selection that significantly differs from individuals not endorsing an MHD?
- What factors predict selection of the multiple call program among tobacco users reporting an MHD?
- Is there a significant difference in 7-month, self-reported quit rates between an Oklahoma sample of MHD and non-MHD tobacco users after using Helpline services?
- Among tobacco users reporting an MHD, are there significant differences in quit rates among those who receive the multiple call program compared to those who receive less intensive service?

The analysis will offer a unique, regional perspective of quitline use in a south-central, high-use, state. The data will be helpful as quitlines make decisions allocating limited resources in a worthwhile effort to maximize their reach and effectiveness with complex, high-risk groups.

## Methods

### OTH programming

The Oklahoma Tobacco Helpline is a free tobacco cessation service available to all residents of Oklahoma and is a program of the Oklahoma Tobacco Settlement Endowment Trust (TSET). Funding for the OTH is primarily provided by TSET, with additional funding provided by the Oklahoma State Department of Health. Residents of Oklahoma can register for OTH services *via* telephone or web, or they can be referred by a health care provider. All registrants are eligible for at least one cessation coaching call and 2 weeks of mono-NRT (either patch, gum, or lozenge) at no cost to the individual. Uninsured, Medicare-, and Medicaid-insured individuals are eligible for a multiple call program of up to five counseling calls and 2–8 weeks of NRT. Prescription medication (e.g., Varenicline, Bupropion) is not available for fulfillment through OTH. Registrants can also enroll into web-, text-, and email-based cessation support services and opt to receive a quit guide mailed to them with written cessation support information.

### Study design and setting

This cohort study was embedded in the overall evaluation of the Oklahoma Tobacco Helpline, and included a retrospective analysis of two cohorts of tobacco users registering for OTH services: those with and without an MHD. Unique OTH registrants from July 2015 to April 2020 were included, as this time period corresponds to the launch of expanded individual services including text, email, web and a two-week NRT starter kit with no required coaching calls. Registrations after April 2020 are not included because of the launch of the expanded behavioral health intervention for tobacco users who report having one or more mental health or substance abuse disorder. The evaluation study includes a 7-month follow-up, with tracking of Helpline services received since baseline registration and an outcome survey of a randomly selected sample of registrants.

### Participant sample

Because we were interested in factors related to engagement and quit rates, participants in this analysis were limited to those eligible for the multiple call program. This included registrants who reported being uninsured, or having Medicaid or Medicare. We retrospectively identified the two groups for comparison. The MHD group was defined as those who reported an MHD by responding affirmatively to a question at registration about diagnosis or treatment for a list of behavioral health and substance abuse conditions. The non-MHD group did not self-identify as having an MHD.

### Data sources

Registration and service utilization data were accessed for those meeting eligibility criteria for this analysis. These data are provided monthly by the quitline provider. Outcome data were obtained from a follow-up survey of a random sample of all OTH registrants. To be eligible for the follow-up evaluation, registrants had to complete at least one intervention call or receive at least 2-weeks of NRT from the OTH. This study includes 7-month follow-up data collected from February 2016 through November 2020, and is limited to randomly selected tobacco users meeting our definitions for the MHD and non-MHD cohorts. Registration and service utilization data are available for 48,770 tobacco users with an MHD and 43,148 tobacco users without an MHD. The follow-up survey sample included 5625 tobacco users with an MHD and 4866 tobacco users without. Response rates for the 7-month follow-up survey were 49.1% for those with an MHD and 50.0% for those without. This study and the overall evaluation of the OTH were reviewed and approved by the University of Oklahoma Health Sciences Center IRB (IRB No. 2616).

### Variables

The following demographic data were collected at registration and used in this analysis: gender (female, male), age (<18, 18–24, 25–44, 45–64, 65+ years), race (White, Black, American Indian, Other), income (< $10,000, $10,000–$19,999, $20,000–$34,999,≥ $35,000), health insurance status (Medicaid, Medicare, and uninsured) and mental health and substance abuse disorder (MHSAD) (none, and 1 or more).

Tobacco use patterns at baseline registration included number of cigarettes per day (none, <20, 20+), frequency of cigarette smoking (daily, non-daily) and time after waking to first cigarette (5, 6–30, 31–60, >60 min). E-cigarette use in the past 30-days at the time of registration was also examined. Mode of quitline registration included phone, online or referral from a health care provider. Type and amount of intervention services received were used to define engagement. They included program (single call program, multiple call program, individual services and WebCoach, an online cessation support platform), number of calls completed (zero, one, two, three or more), and amount of NRT sent by the OTH (no NRT, 2, 4–6, 8+ weeks). An intensity of services (four levels) variable was derived using a combination of the number of calls completed and the amount of NRT shipped to the participants. Supplementary Table 1 displays the combinations of calls and NRT used for each of the four intensity of services levels. All of the levels of intensity of services could also include web and/or text, and/or e-mail.

Quit outcomes were defined using the 7-month follow-up data. We calculated respondent quit rates (30-day point prevalence abstinence) by dividing the number of respondents who reported not smoking in the past 30 days at 7-month follow-up by the total number of respondents to the follow-up survey. Participants in the 7-month follow-up survey were asked if they had at least one quit attempt lasting at least 24-h any time between enrollment and follow-up, regardless of smoking status at the time of the follow-up survey. This was used as a measure of intermediate quit success.

### Statistical methods

We examined and compared the enrollment, engagement and tobacco cessation outcomes among quitline users with one or more MHD to those without an MHD. Descriptive statistics were used to obtain percentages and Pearson chi-square tests were used to test for significant differences between groups. For outcome data gathered through the follow-up survey, we calculated and reported percentages and 95% CIs for each group. We used logistic regression to calculate the odds of selecting the multiple call program among those with an MHD. We used backward selection to identify an adjusted model controlling for confounders. Covariates remained in the adjusted model based on a significance level of 0.1 during model selection. A significance level of 0.05 was used for all final comparisons, and all analyses were conducted using SAS, version 9.4 (SAS Institute Inc., Cary NC).

## Results

The MHD sample was significantly more likely to be female (*p* < 0.0001) and between the ages of 25-44 (*p* < 0.0001, Table 1). They were significantly more likely to report a racial identity of American Indian or Other, and less likely to report identification of Black or White (*p* < 0.0001). Annual income was also significantly lower for MHD-endorsing individuals compared to the non-MHD sample (*p* < 0.0001). Over 80% of those reporting an MHD reported an annual income of < $20,000 (with almost 50% reporting under $10,000). The non-MHD sample was significantly more likely to be uninsured (61.8 vs. 50.8%, *p* < 0.0001), whereas the MHD group was more likely to endorse Medicaid coverage (26.6 vs. 15.4%).

**Table 1:** Characteristics of tobacco users registering for Oklahoma Tobacco Helpline (OTH) services and service utilization, by mental health disorder (MHD) status, July 2015-April 2020, among those eligible for the multiple call program.

**Variable**	**No MHD** ***N** =* **43,148**	**1 or more MHD** ***N** =* **48,770**	***p*** **-value**
	***n*** **(%)**	***n*** **(%)**	
**Sex**			<0.0001
Female	23,417 (54.3)	32,285 (66.2)	
Male	19,720 (45.7)	16,467 (33.8)	
Missing	11	18	
**Age in years**			<0.0001
18–24	3,410 (7.9)	4,246 (8.7)	
25–44	16,662 (38.6)	21,246 (43.6)	
45–64	16,016 (37.1)	19,473 (39.9)	
65+	7,060 (16.4)	3,805 (7.8)	
**Race**			<0.0001
White	30,285 (74.2)	35,062 (73.8)	
Black/African American	4,048 (9.9)	3,750 (7.9)	
American Indian	4,175 (10.2)	5,591 (11.8)	
Other	2,300 (5.6)	3,080 (6.5)	
Missing	2,340	1,287	
**Annual income**			<0.0001
< $10,000	13,459 (35.0)	22,403 (49.8)	
$10,000–19,999	12,792 (33.3)	13,843 (30.8)	
$20,000–34,999	7,696 (20.0)	5,962 (13.3)	
$35,000 +	4,480 (11.7)	2,784 (6.2)	
Missing	4,721	3,778	
**Health insurance**			<0.0001
Medicaid	6,625 (15.4)	12,949 (26.6)	
Medicare	9,862 (22.9)	11,060 (22.7)	
Uninsured	26,661 (61.8)	24,761 (50.8)	
**Cigarettes smoked per day**			<0.0001
<20 per day	18,351 (43.3)	20,142 (41.9)	
20+ per day	24,074 (56.7)	27,904 (58.1)	
Missing	723	724	
**Time to first tobacco**			<0.0001
Within 5 min of waking	20,445 (49.2)	27,053 (56.4)	
6–30 min	14,137 (34.0)	14,512 (30.3)	
31–60 min	4,097 (9.9)	3,724 (7.8)	
>60 min	2,873 (6.9)	2,662 (5.6)	
Missing	1,596	819	
**E-cigarette use in past 30 days**	4,623 (11.8)	8,243 (17.9)	<0.0001
Missing	3,901	2,608	
**Method of registration**			<0.0001
Phone	28,025 (65.0)	33,316 (68.3)	
Web	11,821 (27.4)	8,514 (17.5)	
Referral	3,302 (7.7)	6,940 (14.2)	
**OTH Program enrollment**			<0.0001
Multiple call program	19,036 (44.1)	25,761 (52.8)	
One call program Individual services	132 (0.3) 19,839 (46.0)	279 (0.6) 18,756 (38.5)	
WebCoach	4,141 (9.6)	3,974 (8.1)	
**Intensity of OTH Services received**			<0.0001
1	25,223 (58.5%)	24,831 (51.0%)	
2	5,187 (12.0%)	7,240 (14.8%)	
3	6,659 (15.4%)	8,664 (17.8%)	
4	6,079 (14.1%)	8,035 (16.5%)	

Tobacco use patterns reported by individuals also included some significant differences between groups. The MHD sample was significantly more likely to endorse first tobacco use within 5 min of waking up (56.4 vs. 49.2%, *p* < 0.0001), and were slightly more likely to report smoking over twenty cigarettes daily (58.1 vs. 56.7%, *p* < 0.0001). Those reporting an MHD were significantly more likely to report e-cigarette use in the last 30 days compared to those without MHD (17.9 vs. 11.8%, *p* < 0.0001).

Compared to those without an MHD, individuals endorsing an MHD were significantly more likely to enroll in the comprehensive multiple call program (52.8 vs. 44.1%) and less likely to enroll for individual services (38.5 vs. 46.0%) (*p* < 0.0001, Table 1). They were also more likely to engage with the OTH, with significantly higher rates of service intensity (number of services engaged). 16.5% of persons reporting an MHD received the most intense level of service available. Non-MHD individuals were significantly more likely to enroll in less intensive services (58.5%) such as a 2-week starter kit with no calls with a coach.

Of the 44,797 individuals who enrolled in the multiple call program, a lower proportion of the MHD participants completed no calls (17.9 vs. 21.4%, Table 2). MHD individuals were also significantly more likely to receive 2 weeks of free NRT from the Helpline (17.5 vs. 9.9%, *p* < 0.0001) and less likely to have not received any NRT (24.9 vs. 27.0%). Overall, MHD participants received higher levels of intensity of services within the multiple call program, as compared to those without an MHD.

**Table 2:** Engagement with quitline services by mental health disorder (MHD) status, July 2015-April 2020, among those who enrolled in the multiple call program.

	**No MHD**	**1 or more MHD**	***p*** **-value**
	***N** =* **19,036**	***N** =* **25,761**	
	***n*** **(%)**	***n*** **(%)**	
**Intervention calls completed**			<0.0001
0	4,066 (21.4)	4,610 (17.9)	
1–2	11,596 (60.9)	16,435 (63.8)	
3 +	3,374 (17.7)	4,716 (18.3)	
**NRT sent by the helpline**			<0.0001
No NRT	5,137 (27.0)	6,404 (24.9)	
2 weeks	1,878 (9.9)	4,503 (17.5)	
4–6 weeks	8,277 (43.5)	10,327 (40.1)	
8+ weeks	3,744 (19.7)	4,527 (17.6)	
**Intensity of OTH services received**			<0.0001
1	4,892 (25.7)	5,975 (23.2)	
2	1,441 (7.6)	3,136 (12.2)	
3	6,625 (34.8)	8,615 (33.4)	
4	6,078 (31.9)	8,035 (31.2)	

Multivariable analysis identified several factors associate with the selection of the multiple call program among tobacco users with an MHD and eligible for the service (Table 3). Being female, Black/African American, American Indian, and insured by Medicaid or Medicare were associated with enrollment in the multiple call program compared to less intensive services. Those registering for services as the result of a referral from a healthcare provider or *via* the website had a lower odds of choosing the multiple call program as compared to those registering by phone. Tobacco users who believed their MHD would interfere with quitting at registration had a higher odds of choosing the multiple call program as compared to those who believed their MHD would not interfere. Less addiction, as measured by time to first cigarette at the time of registration, was inversely associated with choosing the multiple call program.

**Table 3:** Predictors of multiple call program enrollment among tobacco users reporting a mental health disorder (MHD) and eligible for the multiple call program [adjusted odds ratios (aOR) and 95% confidence interval (CI)].

**Covariate**	**aOR (95% CI)**	***p*** **-value**
**Sex**		
Female	1.27 (1.22–1.33)	<0.0001
Male	Ref	
**Income**		
< $10,000	Ref	
$10,000–19,999	1.11 (1.06–1.16)	<0.0001
$20,000–34,999	1.19 (1.12–1.27)	
$35,000 +	1.14 (1.04–1.25)	
**Race**		
White	Ref	
Black/African American	1.09 (1.02–1.18)	0.0112
American Indian	1.07 (1.01–1.14)	
Other	1.09 (1.00–1.18)	
**Insurance status**		
Uninsured	Ref	
Medicaid	1.11 (1.05–1.16)	<0.0001
Medicare	1.55 (1.46–1.63)	
**Mode of registration**		
Phone	Ref	
Referral	0.90 (0.85–0.95)	<0.0001
Web	0.60 (0.56–0.63)	
**Belief about role of MHD**		
MHD will not interfere with quitting	Ref	
MHD will interfere with quitting	1.24 (1.18–1.30)	<0.0001
Does not know	0.99 (0.94–1.05)	
**Cigarettes per day**		
<20 per day	Ref	
20+ per day	0.96 (0.92–1.00)	0.0695
**Time to first tobacco**		
Within 5 min of waking	0.87 (0.79–0.96)	0.0002
6–30 min after waking	0.88 (0.79–0.97)	
31–60 min after waking	0.77 (0.69–0.87)	
More than 60 min after waking	Ref	
Used an e-cigarette in the last 30 days	0.98 (0.93–1.04)	0.4662

When assessing quit rates between the MHD vs. non-MHD groups eligible for the multiple call program, response rates at the 7-month follow up call for evaluation for the two groups were very similar (49.1% and 50.0%). Quit rates reported between the two groups did reveal significant differences with the MHD group demonstrating lower quit rates. Responder quit rates were 29.5% for the MHD group vs. 34.1% for the non-MHD group (Table 4). When looking within the MHD group, quit rates differed when assessing quit rates for multiple call program participants vs. less intense program engagement (31.2 and 27.5%).

**Table 4:** Quit outcomes by mental health disorder (MHD) status and program enrollment.

	**At the 7-month follow-up**				
	**Response proportion % (respondents/** **sampled)**	**30-day point-prevalence abstinence % (95% CI)**	***p*** **-value**	**24-h quit attempt % (95% CI)**	***p*** **-value**
**Among those eligible for the multiple call program**					
MHD reported	49.1% (2761/5625)	29.5% (27.8–31.2)	<0.001	86.6% (85.3–87.9)	0.864
No MHD	50.0% (2433/4866)	34.1% (32.3–36.0)		86.8% (85.4–88.1)	
**Among those reporting an MHD**					
Enrolled in multiple call program	52.4% (1536/2929)	31.2% (28.8–33.5)	0.035	87.4% (85.7–89.0)	0.182
Enrolled in less intense services	45.4% (1225/2696)	27.5% (25.0–30.0)		85.6% (83.7–87.6)	

## Discussion

Studies have suggested that individuals with MHD may benefit from *tailored* quitline services to assist them in their attempt to quit; however, there has been limited research on establishing which aspects of current standard quitline service could be changed or augmented to increase quitline effectiveness with this unique population (12, 28). As mentioned previously, this is at least in part due to the fact that the population of individuals endorsing an MHD is not a homogenous group. Furthermore, as seen in the results of this study, significant demographic differences were evident when comparing the MHD and non-MHD groups. The difficult task this presents for researchers and public health agencies is how reasonable adaptation can and should be made in quitline service that uniquely addresses the shared experience of living with an MHD, while simultaneously acknowledging the diverse array of unique attributes represented within an MHD-endorsing group. Due to the sheer number of possible MHDs, their different symptom profiles, etiologies and impacts on functioning, and limited public health resources, effort must be made to explore areas of overlap. To this end, this study sought to not only highlight the unique variables represented in the heterogeneity within an MHD-endorsing quitline group, but to also to contribute to the identification of these cross-cutting, common variables. The goal and dilemma is how to best translate these variables into culturally-relevant, but broadly efficacious, quitline adaptations addressing these overlapping aspects of identity (27).

To our knowledge, this is the largest sample of MHD-endorsing quitline participants that has been examined in the tobacco cessation literature. Overall, it was noteworthy that participants endorsing MHDs tended to engage the more robust treatment option offered at higher rates (multiple call vs. individual services). They also opted to use more of the supplementary supports offered (e.g., text, email, quit guide), as well as accessing at least 2 weeks of free NRT at higher rates. This is consistent with previous findings highlighting the willingness within an MHD sample to accept help with tobacco cessation (29, 30). This is an encouraging finding that supports the use of quitline support as an acceptable option with this unique group.

One question not asked during cessation support *via* the OTH, was whether or not individuals were receiving treatment for their reported MHD or if they had received treatment in the past. Based on the fact that the participant was able to confirm the diagnosis of a specific MHD, it is reasonable to assume that in most, if not all, cases this came as a result of a past interaction with a health care professional who assessed for and determined the presence of the reported diagnosis. Undoubtedly, participants' history of treatment for an MHD prior to quitline engagement varied (i.e., some may have undergone brief or long-term counseling or psychopharmacological treatment for an MHD), but at least one potential explanation for the increased engagement trend observed may be linked to a likely previous history of treatment for MHDs. A willingness to report the presence of an MHD and its potentially complicating impact on a quit attempt also indicates a level of acceptance or acknowledgment of an issue for which the individual needs some assistance. There is internal consistency in the concept that an individual willing to acknowledge that an MHD may negatively impact their quit attempt would also be more willing accept the most supportive service the quitline could offer (as well as have an understanding of their need for additional support). Although this explanation cannot be made definitively, further understanding should be sought for why this group accepts supports offered at higher rates. One potential downside of this style of engagement is that if all available options are accepted and tried at once, it leaves less opportunity for hope (with an unsuccessful quit attempt) that other untapped options may work in the future (31).

As noted above, individuals in the MHD sample were more likely than the non-MHD group to be females in the 25–44-year-old age range, more likely to have an annual income between $10,000 and $20,000 and to be insured by Medicaid. The link between increased willingness to engage in help-seeking and being female, as well as, links between willingness to acknowledge MHDs for females and younger people has been established in previous research (32, 33). A logical connection based off the trends related to income level in this study is that the offer of free NRT (which can be expensive) as well as other free program supports could be more attractive to an individual living with a lower income and unable to pay for support elsewhere.

Consistent with other quitline studies within this group, is that in spite of this higher level of engagement, self-reported quit rates at 7-month follow-up were still significantly lower for the MHD group (21, 22). This follows a well-established trend in the literature that individuals endorsing MHDs report quitting tobacco at lower rates than those without MHDs. One explanation for this finding could be the fact that most, if not all, MHDs are inherently accompanied with (if not defined by) increased difficulties with coping and stress (8, 34). In addition to addressing motivation, a primary feature of many psychotherapeutic approaches is identification of destructive coping patterns and/or identification of more constructive strategies for coping with stress or other unwanted cognitive and behavioral patterns and/or symptoms (35). The process of tobacco cessation requires coping with both the obvious physical impact of nicotine withdrawal, as well as the loss of a, likely long-term habitual, behavior that was very possibly being utilized as a coping mechanism itself.

A plausible hypothesis on the reason lower quit rates persist in spite of more support is that the support is either poorly targeted or insufficient to address the role increased physical and psychological stress plays in thwarting quit attempts. Supporting this hypothesis, Supporting this hypothesis, when Carpenter and colleagues published on their design of a *tailored* quitline approach to specifically help individuals with mental health conditions, it was reported that their cessation support protocol was specifically adapted to increase assessment and attention to a participant's *stress* levels during their quit attempt (12). In-depth explanation for why this was added to the program protocol, other than that it is a typical cause of relapse, and what the stress assessment results were over the course of their pilot were not reported; however, they did report that the program yielded increases in engagement and quit rates among MHD participants in the program. While number of calls and amount of NRT provided was reportedly higher, this study's results don't support a simply “more is better” approach. The protocol design suggested that this population is in need of unique support beyond standard coaching and NRT.

With this emphasis on stress highlighted, the impact of stress on motivation to quit for this unique group should also be considered (36–39). The unique difficulty in achieving high rates of cessation success with this group may be better addressed by offering enhanced support resources and help with the primary stressors both contributing to their tobacco use and acting as a barrier to cessation (19, 40–42). This would likely need to go beyond an assessment of the presence of stress, and to an actual supportive action to connect that individual to a resource that can help them materially or psychologically respond to the stressor in a constructive way. For example, an active resource connection component (such as connection to a 2-1-1 support line or local non-profit) could be integrated into quitline services with a more thorough needs assessment for MHD-endorsing individuals. This could likely be facilitated with a technology-mediated approach in conjunction with a program using ecological momentary assessment to provide real-time options to address stress other than coping by tobacco use (43).

Helpline cessation programs that seek to actively address sources of stress in participants' lives beyond their use of tobacco products will likely incur increased costs due to increased time spent with assessment and connection of individuals to identified resources. The reality of limited funding for many quitlines will require innovative and collaborative solutions to this problem. Future studies should explore the feasibility and efficacy of pairing needs assessment and active resource connection to telephonic and electronic-based tobacco cessation programming. As agencies continue to maintain and market their quitlines as resources, the focus must continue to shift away from an emphasis on “if you build it (and tell them about it), they will come.” The quitline community is encouraged to meaningfully consider why this study found that certain groups demonstrate lower quit rates even when engaging higher amounts of the quitline's services. More pilot studies should be designed examining the impact of incorporating unique support such as stress assessments and amelioration strategies into quitline practice. These studies could provide additional insight into how quitlines can not only help an individual stop a destructive habit but constructively build up new positive habits and supports in its place.

A unique strength of this study was its large sample size of users all eligible for the same OTH service. This allowed for less biased comparisons between groups regarding service selection type and intensity trends in a way that has not before been examined within the literature on tobacco quitlines. An acknowledged limitation of the use of backwards selection for the analysis is that it could have increased the possibility of Type 1 error. This should be taken into account as the results are considered. Although unavoidable due to missing data, variables such as education level, Hispanic ethnicity and sexual orientation would have provided additional information pertinent to the examination and discussion. These variables should be included in future investigations on this topic. It should also be noted that this was a study within a treatment-seeking group of tobacco users. Future comparisons to tobacco users endorsing MHDs not seeking treatment would yield additional insight into potentially helpful strategies for offering the most relevant quitline service. The study was also limited in that MHD status was based solely on self-report. As noted earlier in the discussion, willingness to report MHDs is a study area in itself. As such, it cannot be assumed that the non-MHD comparison group was completely devoid of participants with MHDs (either undiagnosed or unready to share that information).

In conclusion, this study noted the complex reality inherent to tobacco cessation support for individuals dealing with the unique stress of living with mental health difficulties. It supported quitlines as one of the ways this support can be provided, but highlighted the need for unique tailoring, noting that standard quitline care was less effective with this unique group. While this demographically diverse group shares in common the identifier of endorsing an MHD, this is contrasted to the diverse array of symptom presentations within the category of MHDs. This should not, however dissuade the tobacco cessation community from trying to find innovative, impactful ways of attending to the common denominators across this group, to include the role of stress. It is recommended that quitlines and public health entities partner to accomplish this mission. Departments of health and mental health, associations of psychology, counseling, and addiction treatment professionals, public health funders and educational systems should all be sought out as invaluable connections to their region's tobacco quitline. These systems-level partnerships model the universal need for support and provide opportunities for impact multiplication and avoidance of siloed redundancy.

## Data availability statement

The raw data supporting the conclusions of this article will be made available by the authors, without undue reservation.

## Ethics statement

The studies involving human participants were reviewed and approved by University of Oklahoma Health Sciences Center IRB (IRB #2616). Written informed consent for participation was not required for this study in accordance with the national legislation and the institutional requirements.

## Author contributions

JH was responsible for study design, drafting of the introduction, discussion, and article submission. LMB was responsible for statistical analysis. LAB was responsible for study design, methods and results, as well as overseeing statistical analysis and content revision prior to submission. All authors contributed to the article and approved the final version for publication.

## Funding

The Oklahoma Tobacco Settlement Endowment Trust (TSET) is the primary funder of the Oklahoma Tobacco Helpline and also provided funding for the researchers involved in this study.

## Conflict of interest

The authors declare that the research was conducted in the absence of any commercial or financial relationships that could be construed as a potential conflict of interest.

## Publisher's note

All claims expressed in this article are solely those of the authors and do not necessarily represent those of their affiliated organizations, or those of the publisher, the editors and the reviewers. Any product that may be evaluated in this article, or claim that may be made by its manufacturer, is not guaranteed or endorsed by the publisher.
