# Supplementary material for: Unique cessation tools in the box: Quitline utilization and effectiveness trends among a large sample of tobacco users reporting mental health disorders
Source: Front Psychiatry. 2022 Jul 19;13:869802. doi: 10.3389/fpsyt.2022.869802 (PMC9343758; doi:10.3389/fpsyt.2022.869802)
Supplement: Supplementary file 1 [file Table_1.docx]

Supplementary Material

# Supplementary Data

Supplementary Table 1. Intensity of Services: Combination of Calls and Weeks of NRT Shipped

| **Intensity Level** | **Number of completed OTH calls** | **Number of weeks the OTH supplied NRT** |
| --- | --- | --- |
| **Level 1** | 0 | 0-2 |
|  | 1 | 0 |
| **Level 2** | 0 | 4-6 |
|  | 1 | 2 |
|  | 2 | 0 |
| **Level 3** | 0 | 8 or more |
|  | 1 | 4-6 |
|  | 2 | 2 |
|  | >2 | 0 |
| **Level 4** | 1 | 8 or more |
|  | 2 | 4 or more |
|  | >2 | 2 or more |

^a^Intensity of services was derived using a combination of the number of calls completed and the amount of NRT shipped to the participants
